# Differential Synergistic Interactions Among Four Different Wheat-Infecting Viruses

**DOI:** 10.3389/fmicb.2021.800318

**Published:** 2022-01-13

**Authors:** Satyanarayana Tatineni, Jeff Alexander, Feng Qu

**Affiliations:** ^1^United States Department of Agriculture-Agricultural Research Service, Wheat, Sorghum, and Forage Research Unit, Lincoln, NE, United States; ^2^Department of Plant Pathology, University of Nebraska-Lincoln, Lincoln, NE, United States; ^3^Department of Plant Pathology, Ohio Agricultural Research and Development Center, The Ohio State University, Wooster, OH, United States

**Keywords:** barley stripe mosaic virus, brome mosaic virus, co-infection, synergistic interaction, Triticum mosaic virus, virus–virus interaction, wheat, wheat streak mosaic virus

## Abstract

Field-grown wheat (*Triticum aestivum* L.) plants can be co-infected by multiple viruses, including wheat streak mosaic virus (WSMV), Triticum mosaic virus (TriMV), brome mosaic virus (BMV), and barley stripe mosaic virus (BSMV). These viruses belong to four different genera in three different families and are, hence, genetically divergent. However, the impact of potential co-infections with two, three, or all four of them on the viruses themselves, as well as the wheat host, has yet to be examined. This study examined bi-, tri-, and quadripartite interactions among these viruses in wheat for disease development and accumulation of viral genomic RNAs, in comparison with single virus infections. Co-infection of wheat by BMV and BSMV resulted in BMV-like symptoms with a drastic reduction in BSMV genomic RNA copies and coat protein accumulation, suggesting an antagonism-like effect exerted by BMV toward BSMV. However, co-infection of either BMV or BSMV with WSMV or TriMV led to more severe disease than singly infected wheat, but with a decrease or no significant change in titers of interacting viruses in the presence of BMV or BSMV, respectively. These results were in stark contrast with exacerbated disease phenotype accompanied with enhanced virus titers caused by WSMV and TriMV co-infection. Co-infection of wheat by WSMV, TriMV, and BMV or BSMV resulted in enhanced synergistic disease accompanied by increased accumulation of TriMV and BMV but not WSMV or BSMV. Quadripartite interactions in co-infected wheat by all four viruses resulted in very severe disease synergism, leading to the death of the most infected plants, but paradoxically, a drastic reduction in BSMV titer. Our results indicate that interactions among different viruses infecting the same plant host are more complex than previously thought, do not always entail increases in virus titers, and likely involve multiple mechanisms. These findings lay the foundation for additional mechanistic dissections of synergistic interactions among unrelated plant viruses.

## Introduction

Virus–virus interactions of plant viruses can lead to superinfection exclusion (SIE) and synergistic interaction between related and unrelated viruses, respectively (Syller, 2012; Mascia and Gallitelli, 2016; Moreno and Lopez-Moya, 2020). SIE is an antagonistic virus–virus interaction in which initial virus infection prevents subsequent superinfection by closely related viruses (Syller and Grupa, 2016; Zhang et al., 2018). In contrast, synergistic interaction is a facilitative interaction between two or more viruses infecting the same cell; thus, causing mixed infections (Alcaide et al., 2020; Moreno and Lopez-Moya, 2020). Mixed infections with disease synergism drew the attention of growers and researchers due to their impact on plant growth and yield (Fondong et al., 2000; Gutiérrez et al., 2003; Mahuku et al., 2015; Redinbaugh and Stewart, 2018). The advent of high-throughput sequencing technology revolutionized the finding of plants in nature with mixed infections with multiple unrelated viruses to the extent that mixed infections in plants are the norm rather than the exception (Villamor et al., 2019; Minicka et al., 2020; Moreno and Lopez-Moya, 2020). It appears that not all mixed infections in plants are causing a significant effect on plant growth and yield (Moreno and Lopez-Moya, 2020). It is not known why only a certain combination of mixed infections causes severe synergistic disease with drastic effects on plant vigor and productivity.

In mixed infections, the interaction between unrelated viruses can result in disease synergism or neutralism with enhanced and no effect on symptom phenotype, respectively (Alcaide et al., 2020; Moreno and Lopez-Moya, 2020). Mixed infections that result in synergistic interaction can cause severe diseases compared to infections by individual interacting viruses. For example, maize lethal necrosis disease (Mahuku et al., 2015), cassava mosaic disease (Harrison et al., 1997; Fondong et al., 2000), sweet potato virus disease (Gutiérrez et al., 2003), and wheat streak mosaic disease (Tatineni et al., 2010; Byamukama et al., 2012) are the result of interaction between two viruses.

Synergistic interaction is manifested by an increase in virus titer of one or two interacting viruses with an increase in symptom phenotype compared to infections by individual viruses. Mixed infections can cause disease synergism due to an increase in viral replication, viral movement, and interference with host defense mechanisms. Co-infection of tobacco plants by potato virus X (a potexvirus) and a potyvirus (potato virus Y, tobacco etch virus, or plum pox virus) resulted in disease synergism with an increased accumulation of potato virus X but not the potyvirus (Vance, 1991; Vance et al., 1995; Pruss et al., 1997; Shi et al., 1997; González-Jara et al., 2005). In contrast, co-infection by a potyvirus and tobacco mosaic virus or potato spindle tuber viroid resulted in increased accumulation of the potyvirus compared to the other interacting virus or viroid (Valkonen, 1992). In other co-infections, the virus concentration of both interacting viruses will increase with severe disease phenotype (Scheets, 1998; Stenger et al., 2007; Malapi-Nelson et al., 2009; Tatineni et al., 2010). Though the suppressor of RNA silencing proteins of interacting viruses was reported to be involved in synergistic interaction (Ghosh et al., 2021), the mechanisms of these interactions between unrelated viruses for the induction of synergistic disease are not known.

In the Great Plains region of North America, viral diseases can cause 3–5% annual yield losses in wheat, with $76 million yield loss in Kansas alone (Hollandbeck et al., 2017). Agropyron mosaic virus, barley stripe mosaic virus (BSMV), barley yellow dwarf virus, brome mosaic virus (BMV), High Plains wheat mosaic virus, soil-borne wheat mosaic virus, Triticum mosaic virus (TriMV), wheat American striate mosaic virus, and wheat streak mosaic virus (WSMV) are reported to infect wheat in the United States (McKinney and Greeley, 1965; Jensen et al., 1996; Seifers et al., 2008; Hodge et al., 2019). WSMV is one of the most economically important viruses infecting wheat in the Great Plains region of the United States (Brakke, 1987). TriMV was first reported in 2008 from Kansas, followed by several Great Plains states (Seifers et al., 2008; Burrows et al., 2009). Recently, BMV was reported from Ohio wheat fields with incidences up to 25% (Hodge et al., 2019). BSMV primarily infects barley in the field and occasionally infects wheat (Fitzgerald and Timian, 1960; McKinney and Greeley, 1965; Slack et al., 1975; Carroll, 1980).

Wheat streak mosaic virus and TriMV, the type members of *Tritimovirus* and *Poacevirus* genera, respectively, in the family *Potyviridae* (Stenger et al., 1998; Fellers et al., 2009; Tatineni et al., 2009), are transmitted by the wheat curl mite (*Aceria tosichella* Keifer) (Slykhuis, 1955; Seifers et al., 2009; McMechan et al., 2014). The genomes of WSMV and TriMV are single-stranded positive-sense RNAs of 9.4- and 10.2-kb, respectively, encapsulated in flexuous filamentous virus particles. The genomes of both viruses contain a single large open reading frame encoding for large polyproteins of ∼350 kDa that are cleaved into at least 10 mature proteins by three virus-encoded proteinases: P1, HC-Pro, and NIa-Pro (Stenger et al., 1998; Tatineni et al., 2009). Since the wheat curl mite transmits both these viruses, mixed infections with exacerbated disease phenotype and yield loss were common in growers’ fields (Burrows et al., 2009; Byamukama et al., 2012, 2014).

Brome mosaic virus is the type member of the genus *Bromovirus* in the family *Bromoviridae* with a tripartite single-stranded positive-sense RNA genome of RNA1 (3.2 kb), RNA2 (2.9 kb), and RNA3 (2.1 kb) (Kao and Sivakumaran, 2000). These three genomic RNAs of BMV are encapsulated separately by the coat protein (CP) (Rao, 2006). BMV is primarily mechanically transmitted in the field and also vertically transmitted through seed and horizontally by beetles and aphids (Gáborjányi and Szabolcs, 1987; Damsteegt et al., 1992; Mian et al., 2005). Co-infection of WSMV and BMV in triticale was reported in Poland (Trzmiel et al., 2015). BSMV is the type member of the genus *Hordeivirus* in the family *Virgaviridae* with a tripartite genome of RNAα (3.8 kb), RNAβ (3.2 kb), and RNAγ (2.8 kb) (Jackson et al., 2009). Since BMV and BSMV are transmitted by mechanical damage and through seed (Fitzgerald and Timian, 1960; Slack et al., 1975; Edward, 1995; Mian et al., 2005), there is a potential to co-infect wheat with BMV, BSMV, or both with other wheat-infecting viruses.

Previously, synergistic interaction between WSMV and TriMV, two distinct potyvirid species, was examined in wheat (Tatineni et al., 2010, 2019; Byamukama et al., 2012, 2014). However, there is no information on how a wheat-infecting potyvirid interacts with a wheat-infecting non-potyvirid species. We selected mechanically transmissible BMV and BSMV for bi, tri-, and quadripartite interactions with WSMV and TriMV in wheat for studies on disease development and accumulation of viral genomic RNA copies compared to individual virus infections. Antagonism-like synergistic interactions were found between unrelated BMV and BSMV in co-infected wheat with a drastic reduction in BSMV titer. In bi-, tri-, and quadripartite interactions among WSMV, TriMV, BMV, and BSMV in wheat resulted in enhanced synergistic disease with an increase in the number of interacting viruses. This study also revealed that interactions among different unrelated viruses in a co-infected plant host are more complex, and synergistic interactions do not always cause increase in virus titers.

## Materials and Methods

### Virus Isolates

Wheat streak mosaic virus isolate Sidney 81 and TriMV isolate NE were obtained from wheat seedlings cv. Arapahoe infected with *in vitro* transcripts of pSP6-WSMV and pTriMV-R, respectively (Choi et al., 1999; Tatineni et al., 2015). BMV strain M1 was obtained from wheat seedlings infected with *in vitro* transcripts of pB1TP3, pB2TP5, and pB3TP8 (Janda et al., 1987). BSMV strain type was obtained by inoculating wheat seedlings with *in vitro* transcripts of pBSMV-α, pBSMV-β, and pBSMV-γ (Petty et al., 1989). Wheat leaves infected with WSMV, TriMV, BMV, or BSMV were collected at 14 days postinoculation (dpi) and stored at −80°C in 1.0 g aliquots in Ziplock bags until used.

*In vitro* transcripts from pSP6-WSMV-RFP-6K1/CI(7aa) (Tatineni et al., 2016) and pTriMV-GFP-NIb/CP(9aa) (Tatineni et al., 2015) were inoculated onto wheat seedlings at the single leaf stage to obtain WSMV-RFP and TriMV-GFP, respectively. Wheat leaves infected with WSMV-RFP or TriMV-GFP were collected at 14 dpi and stored at −80°C until used.

### Inoculation of Wheat With Single, Double, Triple, and Quadruple Combination of Wheat Streak Mosaic Virus, Triticum Mosaic Virus, Brome Mosaic Virus, and Barley Stripe Mosaic Virus

Wheat cv. Arapahoe seedlings were raised in 15 cm-diameter earthen pots filled with pasteurized soil mix consisting of 34% each of clay loam soil and peat moss, and 16% each of sand and vermiculite. Wheat leaves infected with WSMV, TriMV, BMV, or BSMV were ground in a mortar and pestle in 20 mM sodium phosphate buffer, pH 7.0 (inoculation buffer) at 1:10 dilution (1 g tissue in 9 ml inoculation buffer). Ten ml of WSMV crude sap was mixed with 10 ml of inoculation buffer and 10 ml of TriMV, BMV, or BSMV to obtain 1:30 dilution of WSMV + TriMV, WSMV + BMV, and WSMV + BSMV, respectively. Similarly, 10 ml of TriMV crude sap was mixed with 10 ml of inoculation buffer and 10 ml of BMV or BSMV to obtain TriMV + BMV and TriMV + BSMV, respectively. BMV + BSMV inoculum was obtained by mixing 10 ml each of BMV, BSMV, and inoculation buffer. WSMV + TriMV + BMV or BSMV inocula were obtained by mixing 10 ml of WSMV, TriMV, and BSMV or BSMV, respectively. Inoculum consisting of all four viruses was obtained by mixing equal volumes of WSMV, TriMV, BMV, and BSMV to obtain 1:40 dilution. Virus inocula consisting of one, two, and three viruses were used at 1:30 dilution, and inoculum consisting of all four viruses was used at 1:40 dilution.

Three carborundum-dusted pots of wheat seedlings (12–16 seedlings per pot with 36–48 seedlings per treatment) at the single leaf stage were mechanically inoculated with inoculation buffer (mock) or with crude sap containing one, two, three, or four viruses. Inoculated wheat pots were kept in a greenhouse at 24–26°C maximum and 20–22°C minimum temperature with ∼16 h daylight/supplemental light. Cultural practices and routine pesticide applications were performed to control wheat curl mites and insects on wheat plants. The mock-inoculated wheat plants remained virus-free through the course of this experiment. Wheat seedlings inoculated with virus and mock were observed daily starting at 6 dpi, and symptoms were recorded at 7, 10, 14, 20, and 30 dpi. Symptoms elicited by viruses were photographed at 10, 20, and 30 dpi. The entire experiment was repeated two and three times to determine viral genomic RNA accumulation and symptom phenotype, respectively.

### Isolation of Total RNA

The upper, fully expanded wheat leaves collected at 10, 20, and 30 dpi from the virus- or mock-inoculated wheat plants were used for total RNA extraction. Total RNA was isolated from 200 mg tissue in triplicate from virus-infected and mock-inoculated tissue as described in Tatineni et al. (2010). Briefly, freshly collected wheat leaf pieces were ground into fine powder in liquid nitrogen in a mortar and pestle, followed by the addition of 1.0 ml of TriPure isolation reagent (Roche, Indianapolis, IN, United States). Wheat tissue powder with TriPure reagent was ground thoroughly and the macerate was transferred into 2.0 ml Eppendorf tubes, and 0.25 ml chloroform was added. The tubes were vortexed thoroughly for 30–40 s, followed by incubation at room temperature for 10 min. The tubes were centrifuged at 12,000 *g* for 15 min at 4°C. Total RNA was precipitated from 150 μl of aqueous phase by adding an equal volume of isopropanol, and the tubes were stored at room temperature for 10 min. Total RNA was pelleted by centrifugation at 12,000 × *g* for 10 min at 4°C, and the RNA pellet was washed with 450 μl of 70% ethanol, followed by centrifugation at 12,000 × *g* for 5 min. The RNA pellet was dried in a speed vac for 20 min and suspended in 150 μl sterile water. Total RNA was quantified using a NanoVue Plus Spectrophotometer (GE Healthcare, Buckinghamshire, United Kingdom) and stored at −80°C.

### First-Strand cDNA Synthesis

Total RNA isolated from wheat leaves infected by four individual viruses (WSMV, TriMV, BMV, or BSMV) or co-infections in all combinations (two, three, or four viruses) at 10, 20, and 30 dpi and mock-inoculated wheat leaves were used for first-strand cDNA synthesis. Total RNA (1.0 μg) isolated from virus-infected or mock-inoculated wheat leaves were used for first-strand cDNA synthesis in a 20 μl reaction volume. Each first-strand cDNA reaction consisted of 1X first-strand reaction buffer, 100 μM dNTPs, 2.5 ng/μl random primers (Promega Corporation, Madison, WI, United States), and 8.8 U of avian myeloblastosis virus reverse transcriptase (Roche, Indianapolis, IN, United States). The cDNA synthesis was carried out at 42°C for 1 h and it was followed by termination at 95°C for 5 min. The synthesized cDNA was stored at −20°C until used for real-time PCR.

### Real-Time RT-PCR

Absolute quantification of WSMV, TriMV, BMV, and BSMV genomic RNA copies was determined in 25 μl reaction volumes with TaqMan DNA polymerase (Applied Biosystems) in Applied Biosystems 7300 Real-Time PCR system (Foster City, CA, United States) as described in Tatineni et al. (2010, 2019). Virus-specific primers and probes were used at 500 and 250 nM, respectively. Primers and probes of WSMV, TriMV, BMV RNA1, and BSMV RNAα for real-time PCR (Supplementary Table 1) were synthesized at IDT DNA Technology (Coralville, IA, United States).

The standard amplification protocol provided with the real-time PCR system was used at 50°C for 2 min, 95°C for 15 min, followed by 40 cycles at 95°C for 15 s and 58°C for 60 s. All reactions were performed in triplicate with virus-specific primers and probe for the detection of WSMV, TriMV, BMV, or BSMV. Primers (50 nm) and probe (200 nM) specific to plant 18S rRNA (Applied Biosystems) was used as an internal reference gene. 6-FAM was used as a label for oligonucleotide probes used for the detection of WSMV, TriMV, BMV, and BSMV, while the VIC label was used for oligonucleotide probe used for 18S rRNA.

Standard curves for absolute quantification of WSMV, TriMV, BMV, and BSMV were prepared from serially diluted agarose gel-purified virus-specific PCR fragments. The number of copies in gel-purified PCR fragments that were used to make standard curves was calculated as described in Tatineni et al. (2010).

### Data Analyses and Absolute Quantification of Genomic RNA Copies

The Q-Gene software was used to calculate the absolute number of genomic RNA copies of WSMV, TriMV, BMV, and BSMV in single and mixed infections of wheat (Pfaffl, 2001; Muller et al., 2002; Pfaffl et al., 2002). The Ct value of 18S rRNA of each RNA sample was used for the normalized RNA levels of WSMV, TriMV, BMV, and BSMV. A one-way ANOVA with Tukey HSD was conducted to determine the effect of co-infecting viruses on the accumulation of viral genomic RNA copies.

### Total Protein Isolation and Western Blot Analysis

Total proteins from wheat leaves infected with BMV, BSMV, or BMV + BSMV at 10, 14, and 21 dpi were isolated as described in Tatineni et al. (2011). Five μl of total proteins were analyzed through 4–20% Tris-glycine SDS-polyacrylamide gels (Invitrogen, Carlsbad, CA, United States), followed by Coomassie brilliant blue staining. Ten μl of 1:10 diluted total proteins were separated through 4–20% Tris-glycine SDS-polyacrylamide gels, followed by immunoblot analyses using BMV or BSMV polyclonal antibodies (Agdia, Elkhart, IN, United States) as described in Tatineni et al. (2011).

### Examining the Effect of Brome Mosaic Virus or Barley Stripe Mosaic Virus on the Expression of Wheat Streak Mosaic Virus-RFP and Triticum Mosaic Virus-GFP in Co-infected Wheat

Two pots of wheat cv. Tomahawk seedlings (12–16 seedlings per pot) at the single-leaf stage were inoculated with crude sap of WSMV-RFP, TriMV-GFP, WSMV-RFP + TriMV-GFP, WSMV-RFP + BMV, WSMV-RFP + BSMV, TriMV-GFP + BMV, or TriMV-GFP + BSMV. Virus-inoculated wheat pots were kept in a greenhouse with 16 h daylight/artificial light. The uppermost leaves from 10 different plants were examined under a Stereo Discovery V12 Fluorescence Dissecting Microscope (Carl Zeiss MicroImaging, Inc., NY, United States) using a narrow band of filter set of RFP or GFP, and fluorescent images were obtained with the AxioCam MRc5 camera as described in Tatineni et al. (2016).

## Results

### Brome Mosaic Virus Exerted Antagonism-Like Effect Toward Barley Stripe Mosaic Virus in Co-infected Wheat

**FIGURE 1:**
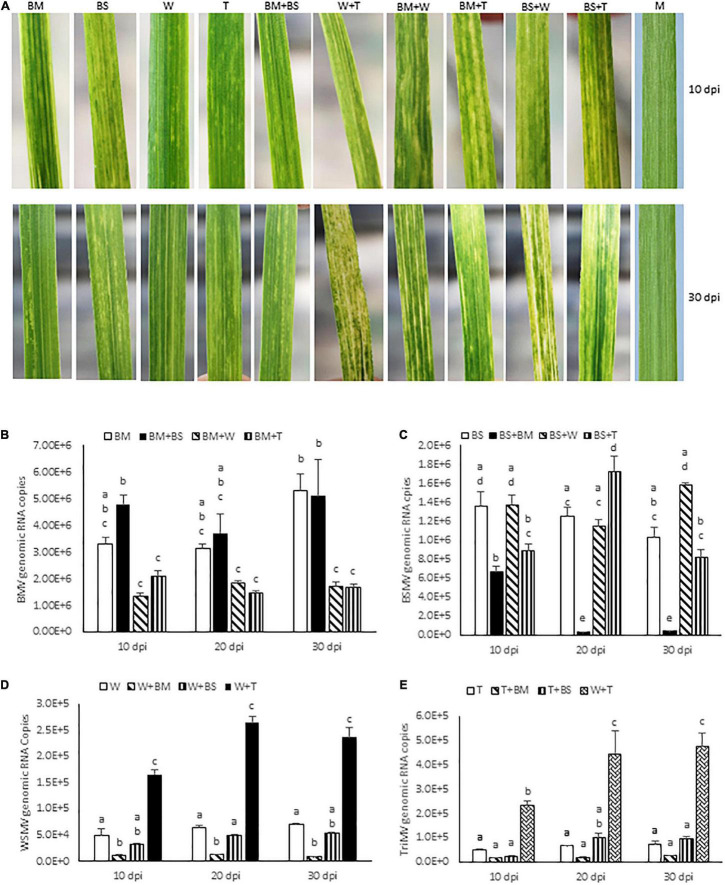
Bipartite synergistic interactions among brome mosaic virus (BMV), barley stripe mosaic virus (BSMV), wheat streak mosaic virus (WSMV), and Triticum mosaic virus (TriMV) in wheat cv. Arapahoe. **(A)** Wheat leaves showing symptoms elicited by BMV, BSMV, WSMV, or TriMV in singly or doubly infected wheat at 10 and 30 days post-inoculation (dpi). Note that co-infection of wheat by BMV and BSMV elicited symptoms similar to those of BMV. **(B–E)** Absolute quantification of genomic RNA copies of BMV **(B)**, BSMV **(C)**, WSMV **(D)**, and TriMV **(E)** in singly or doubly infected wheat at 10, 20, and 30 dpi by real-time RT-PCR. Histograms indicate accumulation of viral genomic RNA copies in 2.5 ng total RNA with standard error. Note that accumulation of BSMV in BMV + BSMV infected wheat was reduced at 20 and 30 dpi compared to BSMV-infected wheat. The same letters above the histograms indicate non-significant, whereas different letters indicate significance at *p* = 0.05. M, Mock-inoculation; BM, BMV; BS, BSMV; W, WSMV; T, TriMV.

**FIGURE 2:**
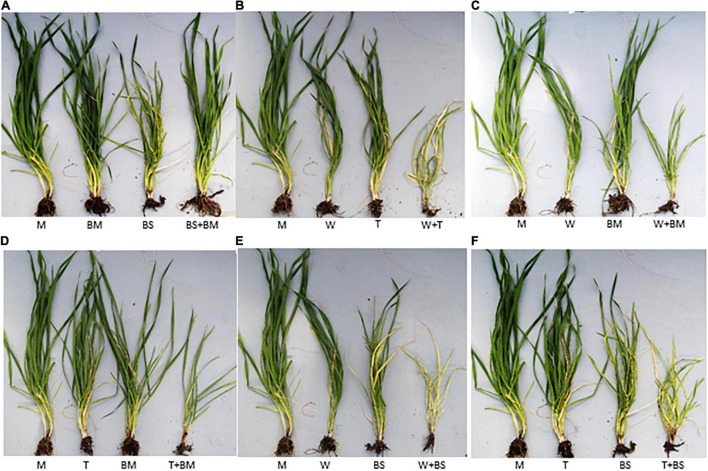
The phenotypic effect of single and double infections of BMV, BSMV, WSMV, and TriMV on wheat cv. Arapahoe at 30 dpi. Phenotypic effects of singly and doubly infected wheat cv. Arapahoe by BMV and BSMV **(A)**, WSMV and TriMV **(B)**, WSMV and BMV **(C)**, TriMV and BMV **(D)**, WSMV and BSMV **(E)**, and TriMV and BSMV **(F)** at 30 dpi. M, mock-inoculation; BM, BMV; BS, BSMV; W, WSMV; T, TriMV.

To examine the interaction between BMV and BSMV, wheat seedlings at the single-leaf stage were inoculated with BMV, BSMV, or BMV + BSMV and the symptom development was monitored. BMV elicited severe chlorotic streaks and spots with mild yellowing of leaves at 7 dpi, followed by moderate chlorotic streaks and mosaic symptoms at 10 dpi (Figure 1A). Subsequently, symptoms on BMV-infected wheat turned into mild chlorotic streaks and mosaic at 20 and 30 dpi (Figure 1A). BSMV also elicited severe chlorotic streaks and patches, which resulted in moderate leaf yellowing at 7–10 dpi (Figure 1A). Symptoms elicited by BSMV turned into mild mosaic, mottling, and chlorotic stripes and patches at 20 dpi. At 30 dpi, plants infected by BSMV had recovered with mild mosaic and mottling with a few leaves showing chlorotic stripes with no apparent leaf yellowing symptoms (Figure 1A). Wheat seedlings co-inoculated with BMV and BSMV developed mild symptoms compared to plants infected with BMV or BSMV at 10 dpi (Figure 1A). At 14 dpi, co-inoculated plants developed BMV-like symptoms with mosaic, mottling, and chlorotic streaks with no leaf yellowing. By 30 dpi, co-infected wheat plants developed mild mosaic, mottling, and chlorotic streak symptoms similar to those in BMV infected wheat (Figure 1A). Co-infection of wheat by BMV + BSMV led to mild stunting, comparable to wheat infected by BMV but unlike the moderately stunted BSMV-infected wheat (Figure 2A), suggesting that co-infection of wheat by unrelated BMV and BSMV did not elicit disease synergism.

#### Barley Stripe Mosaic Virus Genomic RNA Copies and Coat Protein Were Drastically Reduced in Wheat Co-inoculated With Brome Mosaic Virus and Barley Stripe Mosaic Virus

We next examined the effect of co-infection of BMV and BSMV on the accumulation of genomic RNA copies of interacting viruses in wheat at 10, 20, and 30 dpi. BMV genomic RNA copies accumulated at 3.3 × 10^6^, 3.1 × 10^6^, and 5.3 × 10^6^ at 10, 20, and 30 dpi, respectively, in BMV infected wheat (Figure 1B and Table 1). In doubly infected wheat, BMV genomic RNA copies accumulated at similar levels with no statistically significant differences at 10–30 dpi compared to those in BMV-infected wheat (Figure 1B and Table 1). These data suggest that the accumulation of BMV genomic RNA copies was not significantly affected in the presence of BSMV. BSMV genomic RNA copies accumulated at 1.4 × 10^6^, 1.3 × 10^6^, and 1.0 × 10^6^ at 10, 20, and 30 dpi, respectively, in singly infected wheat (Figure 1C and Table 1). In contrast, BSMV genomic RNA copies in BMV + BSMV-infected wheat accumulated at 0. 49-, 0. 03-, and 0.04-fold of BSMV-infected wheat at 10, 20, and 30 dpi, respectively (Figure 1C and Table 1). These data indicated that the presence of BMV caused a drastic reduction in BSMV genomic RNA accumulation.

**TABLE 1:** Absolute quantification of genomic RNA copies of wheat streak mosaic virus (WSMV), Triticum mosaic virus (TriMV), brome mosaic virus (BMV), and barley stripe mosaic virus (BSMV) in single, double, triple, and quadruple infections in wheat^a^.

	10 dpi		20 dpi		30 dpi	
Virus infection	Genomic RNA copies^b^	Fold change^c^	Genomic RNA copies^b^	Fold change^c^	Genomic RNA copies^b^	Fold change^c^
W	49,595 ± 12,890	–	63,005 ± 4,271	–	69,476 ± 3,358	–
W + T	165,524 ± 8,234	3.34***	263,604 ± 11,634	3.76***	236,523 ± 18,463	3.40***
W + BM	10,909 ± 1,805	0.22**	12,118 ± 1,138	0.19**	8,816 ± 321	0.13**
W + BS	31,861 ± 2,406	0.64^ns^	49,446 ± 2,479	0.78^ns^	52,430 ± 3,164	0.75^ns^
W + T + BM	35,114 ± 1,845	0.71^ns^	67,557 ± 2,995	1.07^ns^	57,822 ± 3,007	0.83^ns^
W + T + BS	36,268 ± 2,273	0.73^ns^	85,062 ± 1,235	1.35^ns^	79,677 ± 9,412	1.15^ns^
W + T + BM + BS	26,985 ± 2,015	0.54^ns^	50,119 ± 1,987	0.80^ns^	43,080 ± 3,549	0.62^ns^
T	48,655 ± 3,973	–	67,332 ± 2,178	–	74,066 ± 13,647	–
T + W	234,613 ± 18,458	4.82***	442,012 ± 97,352	6.56***	474,754 ± 57,563	6.41***
T + BM	15,646 ± 1,548	0.32**	18,519 ± 1,767	0.28^ns^	24,353 ± 761	0.33^ns^
T + BS	24,092 ± 1,844	0.50^ns^	101,043 ± 15,822	1.50^ns^	96,030 ± 8,288	1.30^ns^
T + W + BM	70,223 ± 6,160	1.44^ns^	158,825 ± 5,294	2.36^ns^	140,396 ± 16,216	1.90^ns^
T + W + BS	37,865 ± 4,045	0.78^ns^	134,913 ± 2,018	2.00^ns^	164,855 ± 22,020	2.23*
T + W + BM + BS	37,350 ± 8,804	0.77^ns^	106,333 ± 11,791	1.58^ns^	56,638 ± 12,179	0.76^ns^
BM	3,313,111 ± 226,900	–	3,133,176 ± 187,339	–	5,304,346 ± 630,643	–
BM + W	1,323,556 ± 133,917	0.4***	1,846,471 ± 81,308	0.59^ns^	1,696,485 ± 201,072	0.32*
BM + T	2,096,476 ± 200,749	0.63**	1,470,366 ± 75,021	0.47**	1,674,231 ± 108,731	0.32*
BM + BS	4,781,798 ± 360,191	1.44***	3,705,185 ± 718,001	1.18^ns^	5,113,172 ± 1,352,955	0.96^ns^
BM + W + T	7,109,030 ± 298,534	2.15***	8,482,339 ± 511,187	2.71***	13,662,474 ± 1,616,809	2.58***
BM + W + T + BS	5,793,175 ± 256,262	1.75***	6,885,470 ± 156,731	2.2***	6,594,678 ± 848,901	1.24^ns^
BS	1,356,696 ± 151,751	–	1,255,598 ± 91,711	–	1,035,137 ± 97,533	–
BS + W	1,366,185 ± 113,185	1.01^ns^	1,142,213 ± 80,132	0.91^ns^	1,582,425 ± 19,440	1.53***
BS + T	889,908 ± 68,644	0.66**	1,721,571 ± 165,250	1.37**	813,719 ± 91,707	0.79^ns^
BS + BM	671,519 ± 50,075	0.49***	34,697 ± 3,483	0.03***	39,983 ± 5,641	0.04***
BS + W + T	938,207 ± 15,558	0.69**	889,453 ± 64,881	0.71*	1,126,219 ± 191,774	1.09^ns^
BS + W + T + BM	158,418 ± 79,339	0.12***	101,573 ± 45,368	0.08***	38,140 ± 6,887	0.04***

*W, WSMV; T, TriMV; BM, BMV; BS, BSMV; dpi, days post-inoculation.*

*^a^Total RNA extracted from three independent extractions with three replicates each was used for absolute quantification of viral genomic RNA copies with virus-specific primer-probe combination by reverse transcription, followed by real-time polymerase chain reaction.*

*^b^Average number of genomic RNA copies per 2.5 ng of total RNA with standard error (SE). Fold change in genomic RNA accumulation in double, triple, and quadruple mixed infections over single virus infections.*

*^c^REST computer program (Pfaffl et al., 2002) was used to calculate the probability values for differences in accumulation of viruses in mixed infections over single infections of WSMV, TriMV, BMV, or BSMV.*

*, **, ***Represent confidence level at 90, 95, and 99%, respectively. ns, not significant.

**FIGURE 3:**
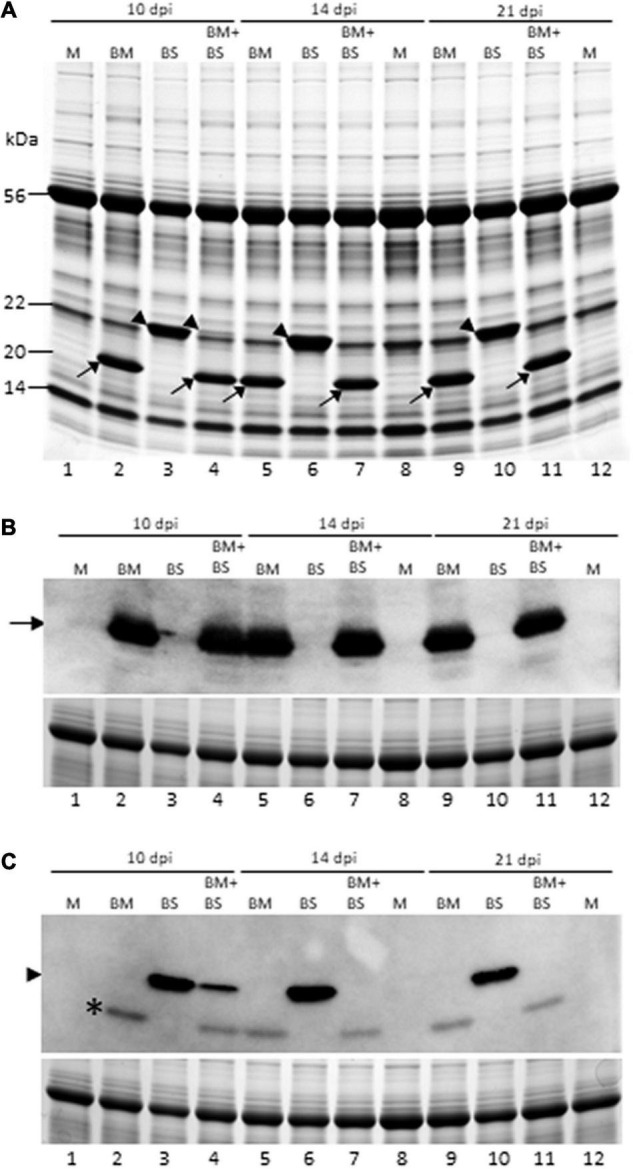
Brome mosaic virus repressed accumulation of BSMV in co-infected wheat. **(A)** Coomassie brilliant blue stained SDS-PAGE gel of total proteins from singly and doubly infected wheat at 10, 14, and 21 dpi. **(B,C)** Western blot analysis of total proteins extracted from wheat leaves infected by BMV, BSMV, or both at 10, 14, and 21 dpi with BMV- **(B)** and BSMV-specific **(C)** polyclonal antibodies. Lower panels **(B,C)** are Coomassie blue-stained SDS-PAGE gels showing the large subunit of wheat RuBisCo protein as a loading control for total protein loaded per well for immunoblot assay. Lanes 1, 8, and 12: mock-inoculated wheat; lanes 2, 5, and 9: BMV-infected wheat; lanes 3, 6, and 10: BSMV-infected wheat; and lanes 4, 7, and 11: BMV + BSMV-infected wheat. Lanes 1–4: 10 dpi; lanes 5–8: 14 dpi; and lanes 9–12: 21 dpi. Location of BMV and BSMV CPs were indicated with arrows and arrowheads, respectively. *The nature of this protein in “C” with BSMV antiserum from BMV-infected wheat is not known.

We next examined the accumulation of BMV and BSMV in singly and doubly infected wheat at 10, 14, and 21 dpi by SDS-PAGE and found that BMV CP accumulated similarly at readily detectable levels in singly and doubly infected wheat (Figure 3A). BSMV CP was also accumulated at readily detectable levels, similar to BMV CP, in singly infected wheat at all the time points (Figure 3A). However, the accumulation of BSMV CP in co-infected wheat drastically decreased at 10 dpi and was undetectable at 14 and 21 dpi compared to BSMV-infected wheat (Figure 3A, lanes 4, 7, and 11). We also examined the accumulation of CPs of BMV and BSMV in singly and doubly infected wheat by immunoblot assay using BMV- and BSMV-specific polyclonal antibodies. BMV CP was accumulated at approximately similar levels in BMV- and BMV + BSMV-infected wheat at all the time points (Figure 3B). In contrast, the accumulation of BSMV CP was substantially reduced in BMV + BSMV-infected wheat at 10 dpi compared to BSMV-infected wheat (Figure 3C, compare lanes 3 and 4). At 14 and 21 dpi, BSMV CP did not accumulate at detectable levels in co-infected wheat (Figure 3C, lanes 7 and 11). These data further confirmed the antagonistic interaction of BMV toward BSMV in co-infected wheat.

### Co-infection of Wheat by Brome Mosaic Virus and Wheat Streak Mosaic Virus or Triticum Mosaic Virus Elicited Disease Synergism With Reduced Accumulation of Interacting Viruses

Previously, we reported that WSMV and TriMV interact synergistically in wheat with severe chlorotic streaks, leaf yellowing, and stunting of plants compared to mild chlorotic streaks, chlorotic spots, mosaic, and mottling symptoms elicited by WSMV or TriMV (Figures 1A, 2B; Tatineni et al., 2010). Synergistic interaction between WSMV and TriMV in wheat resulted in substantially enhanced accumulation of both viruses compared to infections by individual viruses (Figures 1D,E and Table 1; Tatineni et al., 2010, 2019). In this study, we examined how co-infection of wheat by either WSMV or TriMV with BMV or BSMV affected symptom development and genomic RNA accumulation compared to individual virus infections.

#### Co-infection of Wheat by Brome Mosaic Virus and Wheat Streak Mosaic Virus or Triticum Mosaic Virus Elicited Disease Synergism

Wheat co-infected by BMV and WSMV developed severe chlorotic streaks and spots and mild yellowing of leaves at 10 dpi (Figure 1A). At 20–30 dpi, BMV + WSMV-infected wheat developed severe chlorotic streaks with yellow stripes and moderate leaf yellowing and stunting (Figures 1A, 2C). These data revealed that co-infection of wheat by BMV and WSMV elicited severe symptoms compared to mild chlorotic streaks and mosaic symptoms elicited by individual viruses.

Wheat plants co-infected by BMV and TriMV developed chlorotic streaks and spots with severe mosaic and mild leaf yellowing symptoms by 10 dpi (Figure 1A). At 20–30 dpi, BMV + TriMV-infected wheat developed moderate chlorotic stripes and yellow patches with mosaic, mottling, and moderate stunting of plants compared to mild chlorotic streaks and mosaic symptoms elicited by individual viruses (Figures 1A, 2D). Though co-infection of wheat by BMV + TriMV elicited severe symptoms compared to individual virus infections, symptoms elicited by these viruses were slightly milder than those elicited by BMV + WSMV (Figures 1A, 2C,D).

#### Brome Mosaic Virus in Co-infected Wheat With Wheat Streak Mosaic Virus or Triticum Mosaic Virus Reduced the Genomic RNA Copies of Interacting Viruses

We next examined the effect of synergistic interaction between WSMV and TriMV, and BMV and WSMV or TriMV on the accumulation of genomic RNA copies of interacting viruses. Accumulation of WSMV and TriMV genomic RNA copies in wheat co-infected by WSMV + TriMV was increased by 3.3–3.8- and 4.8–6.6-fold of WSMV- or TriMV-infected wheat, respectively, at 10–30 dpi (Figures 1D,E and Table 1). These data confirmed our earlier reports that genomic RNA copies of WSMV and TriMV accumulated at enhanced levels in doubly infected wheat (Tatineni et al., 2010, 2019).

In contrast, genomic RNA copies of WSMV and TriMV were reduced in wheat co-infected with either of these viruses and BMV (Figures 1D,E and Table 1). The genomic RNA copies of WSMV in wheat co-infected by WSMV + BMV were reduced significantly to 0.1–0.2-fold of WSMV-infected wheat at 10–30 dpi (Figure 1D and Table 1). TriMV genomic RNA copies in TriMV + BMV-infected wheat were also reduced but not significantly at 0.3-fold of TriMV-infected wheat at 10–30 dpi (Figure 1E and Table 1). Accumulation of BMV genomic RNA copies in BMV + WSMV or TriMV-infected wheat reduced insignificantly to 0.4–0.6-fold at 10 and 20 dpi and significantly to 0.3-fold at 30 dpi of those in BMV-infected wheat (Figure 1B and Table 1). These data suggest that the BMV titer was slightly reduced at 10 and 20 dpi and significantly reduced at 30 dpi in wheat in the presence of WSMV or TriMV compared to those in BMV-infected wheat. Collectively, these data revealed that the presence of BMV in co-infection with WSMV or TriMV elicited synergistic disease but reduced the titer of interacting viruses.

### Interaction Between Barley Stripe Mosaic Virus and Wheat Streak Mosaic Virus or Triticum Mosaic Virus Elicited Disease Synergism With No Effect on Accumulation of Interacting Viruses

We next examined the interaction between BSMV and WSMV or TriMV in wheat for symptom development and accumulation of genomic RNA copies of interacting viruses. Wheat co-infected by BSMV and WSMV or TriMV developed chlorotic streaks and mild leaf yellowing symptoms similar to BSMV infection at 10 dpi (Figure 1A). In contrast to the recovery of BSMV-infected wheat at 20 dpi, wheat co-infected by BSMV and WSMV or TriMV developed severe chlorotic streaks and leaf yellowing symptoms with a few leaves showing bleaching, leaf narrowing, and stunting of plants by 30 dpi (Figures 1A, 2E,F). Wheat co-infected by BSMV + WSMV developed more pronounced stunting and leaf yellowing symptoms compared to BSMV + TriMV infected wheat at 30 dpi (Figures 1A, 2E,F).

The genomic RNA copies of WSMV accumulated in BSMV + WSMV-infected wheat at 0.6–0.8-fold of WSMV-infected wheat at 10–30 dpi (Figure 1D and Table 1), suggesting that WSMV accumulation was reduced slightly but not significantly in the presence of BSMV. The genomic RNA copies of TriMV in the presence of BSMV in co-infected wheat accumulated at 0.5–1.5-fold of TriMV-infected wheat at 10–30 dpi (Figure 1E and Table 1). Accumulation of BSMV genomic RNA copies in BSMV + WSMV infected wheat was at 0.9–1.5-fold of BSMV-infected wheat at 10–30 dpi, suggesting that BSMV titer was not significantly affected in wheat in the presence of WSMV (Figure 1C and Table 1). However, accumulation of BSMV genomic RNA copies in BSMV + TriMV infected wheat was significantly decreased (0.7-fold) and increased (1.4-fold) at 10 and 20 dpi, respectively, but no significant change (0.8-fold) at 30 dpi compared to those in BSMV-infected wheat (Figure 1C and Table 1).

### Tripartite Interactions Between Wheat Streak Mosaic Virus, Triticum Mosaic Virus, and Brome Mosaic Virus or Barley Stripe Mosaic Virus Elicited Severe Disease Synergism in Wheat

**FIGURE 4:**
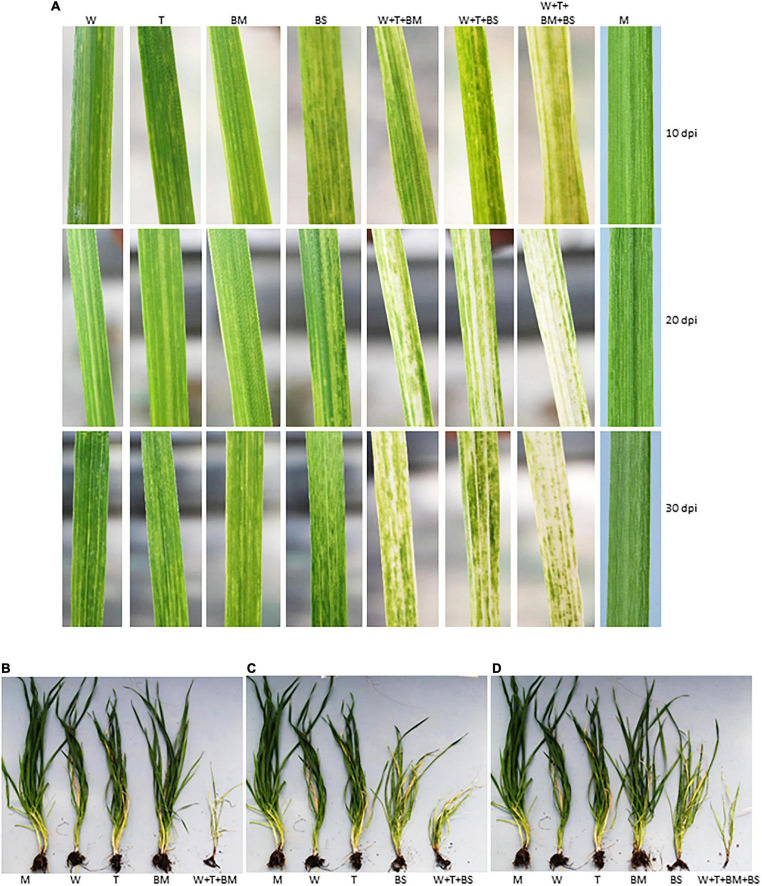
Tri- and tetrapartite synergistic interactions among WSMV, TriMV, BMV, and BSMV in wheat cv. Arapahoe. **(A)** Wheat leaves showing the symptom phenotype of individual and co-infections by WSMV, TriMV, and BMV or BSMV or both on wheat at 10, 20, and 30 dpi. Note that co-infection of wheat by BMV or BSMV with WSMV and TriMV elicited more severe symptoms compared to individual virus infections. Co-infection of wheat by all four viruses induced severe chlorosis and bleaching of leaves. **(B–D)** Effect of co-infection of wheat by triple [WSMV + TriMV + BMV **(B)** and WSMV + TriMV + BSMV **(C)**] or quadruple **(D)** viruses on wheat phenotype compared to singly infected plants. Wheat seedlings at the single leaf stage were inoculated singly or with a combination of viruses, and wheat plants at 30 dpi were uprooted and photographed. M, mock inoculation; W, WSMV; T, TriMV; BM, BMV; BS, BSMV.

The effect of synergistically interacting WSMV and TriMV in combination with BMV or BSMV on disease development was examined by co-inoculating wheat with WSMV, TriMV, and BMV or BSMV. At 7 dpi, wheat seedlings inoculated with WSMV + TriMV + BMV developed chlorotic streaks and spots similar to symptoms elicited by individual viruses. However, at 10 dpi, wheat co-infected by three viruses developed severe chlorotic streaks, mosaic, and yellowing of leaves compared to mild symptoms elicited by individual viruses (Figure 4A). WSMV + TriMV + BMV elicited severe chlorotic streaks in co-infected wheat resulting in large yellow patches turning the leaves into mild bleaching at 20 dpi, followed by severe stunting with yellowing of leaves and tiller deformation at 30 dpi (Figures 4A,B). The presence of BMV with WSMV and TriMV in wheat elicited more severe symptoms compared to any dual infection combination of WSMV, TriMV, and BMV.

The presence of BSMV in combination with WSMV and TriMV also elicited severe disease synergism with severe chlorotic streaks, mosaic, and leaf narrowing and yellowing symptoms by 20 dpi (Figure 4A). At 30 dpi, most of the leaves developed mild bleaching due to severe chlorotic streaks and patches with severe stunting of plants (Figures 4A,C). These data suggest that the presence of BMV or BSMV with WSMV and TriMV caused additive synergism that resulted in enhanced disease phenotype compared to co-infection by WSMV and TriMV.

**FIGURE 5:**
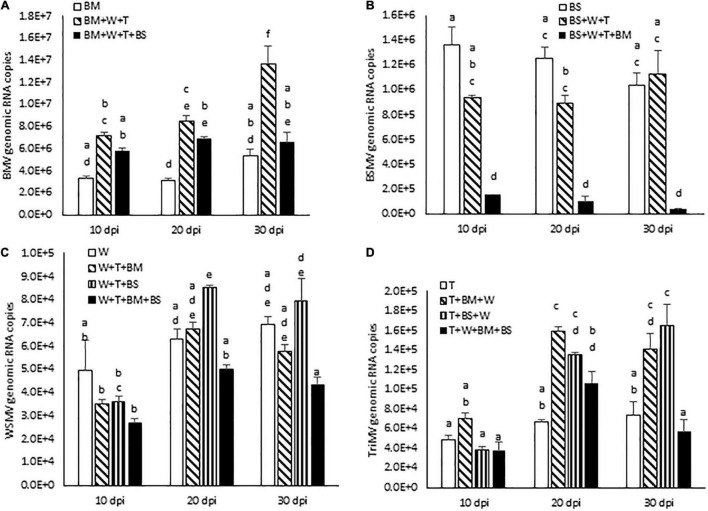
Effect of co-infection of wheat by three (WSMV, TriMV, and BMV or BSMV) or four viruses (WSMV, TriMV, BMV, and BSMV) on genomic RNA accumulation of interacting viruses at 10, 20, and 30 dpi. Absolute quantification of genomic RNA copies of BMV **(A)**, BSMV **(B)**, WSMV **(C)**, and TriMV **(D)** were determined by real-time RT-PCR. Histograms indicate accumulation of viral genomic RNA copies in 2.5 ng total RNA with standard error. The same letters above the histograms indicate non-significant, whereas different letters indicate significance at *p* = 0.05. BM, BMV; BS, BSMV; W, WSMV; T, TriMV.

We next examined the effect of increased disease synergism of WSMV and TriMV in the presence of BMV or BSMV on the accumulation of genomic RNAs of interacting viruses. The presence of WSMV and TriMV caused a significant increase in BMV genomic RNA accumulation by 2.2–2.7-fold of BMV-infected wheat at 10–30 dpi (Figure 5A and Table 1). In contrast, BSMV genomic RNA copies in wheat in the presence of WSMV and TriMV accumulated at 0.7–1.1-fold of BSMV-infected wheat (Figure 5B and Table 1), suggesting that BSMV titer was not significantly changed in the presence of WSMV and TriMV. TriMV genomic RNA copies in TriMV + WSMV + BMV or BSMV- infected wheat were significantly increased by 1.9–2.4-fold of TriMV-infected wheat at 20 and 30 dpi (Figure 5D and Table 1). In contrast, no significant change was found in the accumulation of WSMV in TriMV + WSMV + BMV or BSMV-infected wheat as WSMV genomic RNA copies accumulated at 0.7–1.4-fold of WSMV-infected wheat at 10–30 dpi (Figure 5C and Table 1).

### Effect of Co-infection by Quadruple Viruses on Disease Synergism in Wheat

We next examined quadripartite interactions among BMV, BSMV, WSMV, and TriMV in wheat on disease synergism and accumulation of genomic RNA copies of interacting viruses. Wheat seedlings inoculated with a mixture of four viruses developed severe chlorotic streaks, mosaic, yellowing, and leaf narrowing symptoms by 10 dpi (Figure 4A), followed by severe leaf chlorosis with pronounced leaf deformation and bleaching at 14 dpi. At 20 dpi, wheat plants co-infected by four viruses developed severe leaf bleaching (Figure 4A), and ∼20% of infected plants were dead. By 30 dpi, infection of wheat by four viruses elicited severe chlorosis and stunting, resulting in the death of most infected plants (Figures 4A,D).

We next examined the effect of severe synergism in wheat co-infected by all four viruses on the accumulation of genomic RNA copies of interacting viruses. WSMV and TriMV genomic RNA copies in wheat co-infected by four viruses accumulated at 0.5–0.8- and 0.8–1.6-fold of WSMV- or TriMV-infected wheat, respectively, at 10–30 dpi (Figures 5C,D and Table 1). These data suggest that WSMV and TriMV titer in wheat co-infected by four viruses did not change significantly compared to WSMV or TriMV infected wheat. BMV genomic RNA copies in co-infected wheat by four viruses accumulated at 1.2–2.2-fold of BMV-infected wheat at 10–30 dpi (Figure 5A and Table 1). In contrast, the accumulation of BSMV genomic RNA copies in wheat co-infected by four viruses was reduced drastically to 0.04–0.12-fold of BSMV-infected wheat at 10–30 dpi (Figure 5B and Table 1).

### Brome Mosaic Virus and Barley Stripe Mosaic Virus Affected the Expression of Wheat Streak Mosaic Virus and Triticum Mosaic Virus in Co-infected Wheat

**FIGURE 6:**
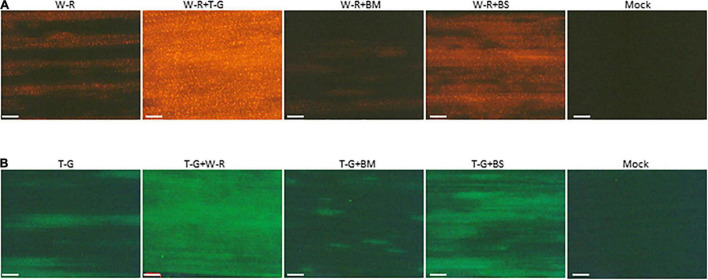
Brome mosaic virus and BSMV affected the expression of WSMV and TriMV in co-infected wheat. Wheat seedlings at the single-leaf stage were inoculated with WSMV-RFP, TriMV-GFP, WSMV-RFP + TriMV-GFP, WSMV-RFP + BMV, WSMV-RFP + BSMV, TriMV-GFP + BMV, or TriMV-GFP + BSMV. Fluorescent images of wheat leaves infected singly or doubly in combination with WSMV-RFP or TriMV-GFP were observed with an RFP filter **(A)** or GFP filter **(B)**, respectively, under a Stereo Discovery V12 fluorescent microscope at 14 days post-inoculation. W-R, RFP-tagged WSMV (WSMV-RFP); T-G, GFP-tagged TriMV (TriMV-GFP); BM, BMV; BS, BSMV. Note reduced fluorescent protein in wheat co-infected by BMV and WSMV-RFP or TriMV-GFP. Compare images W-R + BM with W-R + T-G and T-G + BM with T-G + W-R. Bars represent 500 μm.

The above data revealed that interaction between BMV and WSMV or TriMV caused synergism with a decrease in accumulation of WSMV (significantly) and TriMV (not significantly) compared to individual infections. To further examine whether the presence of BMV or BSMV in co-infected wheat has any effect on the expression of WSMV and TriMV, an RFP-tagged WSMV or GFP-tagged TriMV was used to co-infect wheat with BMV or BSMV. At 14 dpi, expression of RFP was substantially reduced in wheat co-infected by WSMV-RFP + BMV compared to those of WSMV-RFP + TriMV-GFP or WSMV-RFP (Figure 6A), suggesting that expression of WSMV was reduced in the presence of BMV. Expression of RFP in wheat co-infected by WSMV-RFP + BSMV was increased compared to WSMV-RFP-infected wheat, but substantially less compared to wheat co-infected by WSMV-RFP + TriMV-GFP (Figure 6A). Expression of GFP in wheat co-infected by TriMV-GFP and BMV was substantially less compared to that of WSMV-RFP + TriMV-GFP- or TriMV-GFP-infected wheat (Figure 6B). However, GFP expression in wheat co-infected by TriMV-GFP and BSMV was higher compared to that of TriMV-GFP-infected wheat but less than WSMV-RFP + TriMV-GFP infected wheat (Figure 6B). Taken together, these data revealed that the presence of BMV in co-infected wheat reduced the expression of WSMV or TriMV but still resulted in disease synergism.

## Discussion

Synergistic interactions between two unrelated viruses in plants have been examined extensively for several virus combinations (Alcaide et al., 2020; Moreno and Lopez-Moya, 2020). However, information on the interaction between more than two unrelated viruses is scanty. In this study, we examined bi-, tri-, and quadripartite interactions among WSMV, TriMV, BMV, and BSMV in wheat. BMV exerted antagonistic-like interaction toward unrelated BSMV in co-infected wheat. However, co-infection of either BMV or BSMV or both with WSMV or TriMV or both in bi-, tri-, and quadripartite interactions developed enhanced disease synergism with the increased number of interacting viruses and differential effects on titers of interacting viruses.

Mixed infections by unrelated viruses would lead to synergism with enhanced disease or neutralism with no effect on disease compared to infections by individual viruses (Alcaide et al., 2020; Moreno and Lopez-Moya, 2020). In contrast, antagonism-like interaction was observed between unrelated BMV and BSMV in co-infected wheat. Generally, antagonistic interactions were observed between the strains of the same virus but not between unrelated viruses (Syller, 2012; Zhang et al., 2018). Wheat co-infected by BMV and BSMV developed BMV-like symptoms with a drastic reduction in BSMV genomic RNA copies and CP compared to those in BSMV-infected wheat. In contrast, no significant change in BMV genomic RNA copies and CP was observed in BMV + BSMV-infected wheat. These data revealed that BMV suppressed the replication and expression of BSMV-encoded genes in co-infected wheat. Antagonistic interactions between unrelated papaya ringspot virus (PRSV, a potyvirus) and papaya mosaic virus (PapMV, a potexvirus) were observed only when papaya plants were previously infected by PapMV (Chávez-Calvillo et al., 2016). But synergistic interaction with increased disease phenotype was observed when papaya plants were prior infected by PRSV or co-infected by PRSV and PapMV; thus, it differs from the antagonism-like interaction observed between BMV and BSMV in co-infected wheat.

Previously, we reported disease synergism in co-infected wheat by WSMV and TriMV with a substantial increase in titers of both interacting viruses (Tatineni et al., 2010, 2019). In bipartite interactions, BMV or BSMV interacted synergistically with WSMV or TriMV. However, the presence of BMV reduced the titer of WSMV (significantly) and TriMV (not significantly), while BSMV had no significant effect. These data suggest that though the titer of interacting viruses decreased or remained unchanged in co-infected wheat, the interaction between a potyvirid species (WSMV or TriMV) and a non-potyvirid species (BMV or BSMV) caused disease synergism. These results also revealed that in contrast to earlier studies on synergistic interactions (Vance, 1991; Valkonen, 1992; Scheets, 1998; Stenger et al., 2007; Malapi-Nelson et al., 2009; Tatineni et al., 2010), mixed infections with reduced titers of both interacting viruses could also elicit disease synergism. Though BMV and BSMV in co-infected wheat did not elicit disease synergism, the presence of WSMV or TriMV in combination with BMV or BSMV elicited synergism, suggesting that WSMV and TriMV encoded proteins were involved in a synergistic interaction with BMV or BSMV.

In tripartite interactions, the presence of either BMV or BSMV with WSMV and TriMV elicited enhanced disease synergism compared to co-infection by WSMV and TriMV. Co-infection of wheat by WSMV and TriMV caused enhanced titers of both interacting viruses (Tatineni et al., 2010). However, the presence of BMV or BSMV with WSMV and TriMV caused no significant change in WSMV titer and enhanced TriMV titer, suggesting that BMV and BSMV interacted similarly in tripartite interactions with TriMV and WSMV. The presence of WSMV and TriMV with BMV or BSMV caused enhanced and no significant change in BMV and BSMV titers, respectively, suggesting that WSMV and TriMV differentially interacted with BMV or BSMV in tripartite interactions in wheat.

Of all the interactions examined in this study, the quadripartite interactions in wheat co-infected by all four viruses caused the most severe synergism, with severe stunting and death of most plants. These data suggest that the presence of BMV and BSMV with WSMV and TriMV caused additive effects on synergistic interactions that resulted in lethal synergistic disease. Quadripartite interactions in wheat resulted in no significant change in WSMV and TriMV titers, increased BMV titer, and a drastic reduction in BSMV titer. Accumulation of BSMV genomic RNA copies did not change significantly in BSMV co-infected with WSMV and TriMV, but the addition of BMV to BSMV, WSMV, and TriMV resulted in an 8- to 25-fold decrease in BSMV titer. These data further confirm that the presence of BMV in co-infected wheat affected BSMV replication.

Antagonistic-like interactions between unrelated BMV and BSMV in co-infected wheat resulted in a 2- to 33-fold decrease in BSMV titer. In synergistic interactions, suppressors of RNA silencing proteins of interacting viruses play an important role in causing the disease synergism (Vance et al., 1995; Shi et al., 1997; González-Jara et al., 2005; Ghosh et al., 2021). The γb protein of BSMV has been reported as a suppressor of RNA silencing (Bragg and Jackson, 2004), but a suppressor of RNA silencing protein has not been identified conclusively for BMV (He et al., 2021). The lack of synergistic interaction between BMV and BSMV in co-infected wheat might be due to the lack of BMV-encoded RNA silencing suppressor. It is not clear how BMV represses the BSMV replication in co-infected wheat. Perhaps, the robust replication nature of BMV outcompetes BSMV for host resources; thus, preventing BSMV replication, movement, or both. Alternatively, the interaction between BMV and BSMV and host factors might be high-temperature dependent, or BMV proteins interfere with or block BSMV and host protein interactions.

In mixed infections, efficient replication of one of the interacting viruses likely plays an important role in determining the titers of other interacting partner viruses. In singly infected wheat, BMV accumulated at 50-76-, 47-72-, and 2.4-5.1-fold more than WSMV, TriMV, and BSMV, respectively. BSMV accumulated at 15-27-, 14-28-, and 0.2-0.4-fold of WSMV, TriMV, and BMV, respectively, in singly infected wheat. These data further support that the robust replication of tripartite BMV in wheat might have depleted the shared resources for replication of another tripartite virus. Interestingly, BMV repressed BSMV accumulation but not WSMV and TriMV in quadripartite interactions. The robust replication of BMV might have also depleted host resources for moderately replicating WSMV or TriMV in mixed infections in wheat; thus, BMV caused decreased genomic RNA accumulation and gene expression of WSMV and TriMV in bipartite interactions. However, efficient suppressors of RNA silencing of WSMV (Young et al., 2012) and TriMV (Tatineni et al., 2012) in mixed infections with BMV or BSMV or both might have caused the disease synergism. Additionally, the presence of synergistically interacting WSMV and TriMV in tripartite and quadripartite interactions might have prevented the negative effects of BMV on WSMV and TriMV. Perhaps, the weak nature of RNA silencing suppressor protein of BSMV (Yelina et al., 2002; Bragg and Jackson, 2004; Zhang et al., 2017) is not enough to overcome the host defense and compete with robust BMV replication; thus, BMV dominates replication of BSMV in co-infected wheat.

Previously, we found that during the early stages of synergistic interaction between WSMV and TriMV, prior infection of wheat by TriMV facilitated the accelerated long-distance movement of WSMV (Tatineni et al., 2019). In contrast, systemic infection of TriMV was delayed in WSMV-infected wheat, suggesting that some of the TriMV-encoded proteins are involved in synergistic interaction between WSMV and TriMV. Similarly, some of the virus-encoded proteins of interacting viruses may also be involved in bi-, tri-, and quadripartite synergistic interactions among WSMV, TriMV, BMV, and BSMV in wheat. Our data also suggest that interactions between four viruses are complex in wheat, and the presence of multiple interacting viruses in a host might have also affected the dynamics of virus-host interactions: thus, leading to differential accumulation of interacting viruses. Further studies are required to delineate the mechanisms behind atypical disease synergism in bi-, tri-, and quadripartite interactions among WSMV, TriMV, BMV, and BSMV in wheat.

## Data Availability Statement

The original contributions presented in the study are included in the article/Supplementary Material, further inquiries can be directed to the corresponding author.

## Author Contributions

ST formulated and designed the experiments and wrote the manuscript. ST and JA performed the research. ST, FQ, and JA analyzed the data, edited the manuscript, and approved the submitted version.

## Author Disclaimer

USDA is an equal opportunity provider and employer. Mention of trade names or commercial products in this publication is solely for the purpose of providing specific information and does not imply recommendation or endorsement by the U.S. Department of Agriculture.

## Conflict of Interest

The authors declare that the research was conducted in the absence of any commercial or financial relationships that could be construed as a potential conflict of interest.

## Publisher’s Note

All claims expressed in this article are solely those of the authors and do not necessarily represent those of their affiliated organizations, or those of the publisher, the editors and the reviewers. Any product that may be evaluated in this article, or claim that may be made by its manufacturer, is not guaranteed or endorsed by the publisher.
