# Supplementary material for: Differential Synergistic Interactions Among Four Different Wheat-Infecting Viruses
Source: Front Microbiol. 2022 Jan 13;12:800318. doi: 10.3389/fmicb.2021.800318 (PMC8793356; doi:10.3389/fmicb.2021.800318)
Supplement: Supplementary file 1 [file Table_1.DOCX]

| Table S1. Nucleotide sequences of forward and reverse primers and probes used for real-time PCR quantification of genomic RNAs of wheat streak mosaic virus (WSMV), Triticum mosaic virus (TriMV), brome mosaic virus (BMV), and barley stripe mosaic virus (BSMV). | | | |
| --- | --- | --- | --- |
|  |  |  |  |
|  |  |  |  |
| Primer | Nucleotide sequence (5'-3') | | Position |
| WSMV-F | AAGTGCAGAACAGCGTTG | | 9075-9092^a^ |
| WSMV-R | AAACTGTGCGTGTTCTCC | | 9213-9196 |
| WSMV-Probe | ACTGAGTGCGGGTACTAATGAGGAC | | 9142-9166 |
| TriMV-F | CTTTGGAGCTTTAGCCTACC | | 9754-9773^b^ |
| TriMV-R | CTGGTCCACTGTCACAAA |  | 9892-9875 |
| TriMV-Probe | CAAGCCAATTATACCGCACAACGC | | 9802-9825 |
| BMV RNA1-F | CTCGAAGAAGAAGGCGAAAGT | | 1592-1612^c^ |
| BMV RNA1-R | AGTCACCGGTTTAACGTCATC | | 1706-1687 |
| BMV RNA1-Probe | ACCTCAGGAGGAGTTTCATGATGCC | | 1631-1655 |
| BSMV RNAα-F | CCAGAGCTGAGTTTCTCTACTATC | | 2148-2171^d^ |
| BSMV RNAα-R | CAATGGAACGGCCACTTTATC | | 2269-2249 |
| BSMV RNAα-Probe | AGCGTGATTTGTGAAAGGGCTCAGA | | 2179-2204 |
| The sequence of primers and probes for WSMV and TriMV was reported in Tatineni et al. (2010).  Nucleotide positions correspond to the genomic RNA sequence of TriMV^a^ (GenBank accession # FJ669487), WSMV^b^ (GenBank accession # AF057533), BMV RNA1^c^ (GenBank accession # X02380), and BSMV RNAα^d^ (GenBank accession # J04342). | | | |
|  |  |  |  |
|  | | | |
|  |  |  |  |
